# Socioeconomic disparities in Korea by health insurance type during the COVID-19 pandemic: a nationwide study

**DOI:** 10.4178/epih.e2021007

**Published:** 2021-01-13

**Authors:** Han Eol Jeong, Jongseong Lee, Hyun Joon Shin, Ju-Young Shin

**Affiliations:** 1 School of Pharmacy, Sungkyunkwan University, Suwon, Korea; 2 Columbia School of Social Work, Columbia University, New York, NY, USA; 3 Lemuel Shattuck Hospital, Boston, MA, USA; 4 Brigham and Women’s Hospital, Boston, MA, USA; 5 Department of Clinical Research Design and Evaluation, Samsung Advanced Institute for Health Sciences and Technology (SAIHST), Sungkyunkwan University, Seoul, Korea

**Keywords:** COVID-19, Socioeconomic disparities, Health insurance type

## Abstract

**OBJECTIVES:**

This study explored socioeconomic disparities in Korea using health insurance type as a proxy during the ongoing coronavirus disease 2019 (COVID-19) pandemic.

**METHODS:**

We conducted a retrospective cohort study using Korea’s nationwide healthcare database, which contained all individuals who received a diagnostic test for COVID-19 (n=232,390) as of May 15, 2020. We classified our cohort by health insurance type into beneficiaries of the National Health Insurance (NHI) or Medicaid programs. Our study outcomes were infection with severe acute respiratory syndrome coronavirus 2 (SARS-CoV-2) and COVID-19-related outcomes, a composite of all-cause death, intensive care unit admission, and mechanical ventilation use. We estimated age-, sex-, and Charlson comorbidity index score–adjusted odds ratios (aORs) with 95% confidence intervals (CIs) using a multivariable logistic regression analysis.

**RESULTS:**

Of the 218,070 NHI and 14,320 Medicaid beneficiaries who received COVID-19 tests, 7,777 and 738 tested positive, respectively. The Medicaid beneficiaries were older (mean age, 57.5 vs. 47.8 years), more likely to be males (47.2 vs. 40.2%), and had a higher comorbidity burden (mean CCI, 2.0 vs. 1.7) than NHI beneficiaries. Compared to NHI beneficiaries, Medicaid beneficiaries had a 22% increased risk of SARS-CoV-2 infection (aOR, 1.22; 95% CI, 1.09 to 1.38), but had no significantly elevated risk of COVID-19-related outcomes (aOR 1.10, 95% CI 0.77 to 1.57); the individual events of the composite outcome yielded similar findings.

**CONCLUSIONS:**

As socioeconomic factors, with health insurance as a proxy, could serve as determinants during the current pandemic, pre-emptive support is needed for high-risk groups to slow its spread.

## INTRODUCTION

Socioeconomic inequalities are a prominent reason for health disparities [1,2], as they may profoundly impact the incidence of disease and its treatment, ultimately exacerbating health inequality [3]. Thus, many countries have a sub-system within their health insurance program, such as Medicaid, in which the government provides financial support depending on an individual’s income or socioeconomic status. However, it remains uncertain as to whether these systems help mitigate health disparities or disease incidence among beneficiaries, especially during times of economic or epidemic crises.

With mounting evidence of associations between socioeconomic status and disease incidence [4-6], individuals residing in deprived environments were found to have a higher prevalence of infectious diseases than their counterparts [7,8]. In Korea, the number of health insurance claims for non-communicable diseases per Medicaid beneficiaries (low-income families; i.e., individuals eligible for the National Basic Living Security Act) was nearly quadruple than that for national health insurance beneficiaries [9]. Beneficiaries of the Medicaid program accounted for approximately 3% of all Koreans, with the remaining population covered by the National Health Insurance (NHI) program [9]. Moreover, chronic conditions of diabetes mellitus or hypertension were associated with poor clinical outcomes among those with infectious diseases [10], while prolonged hospitalizations with greater exposure to causative pathogens may increase the incidence of infectious diseases [11].

Socioeconomic inequalities continue to widen the gap in healthcare accessibility among patients with infectious diseases, which may heighten health inequality. Relative to private insurance beneficiaries, Medicaid beneficiaries were estimated to be more likely to experience death but to receive less costly treatments due to their financial burden [3,12,13]. Socioeconomic status may explain health inequalities in clinical outcomes, as well as variation in disease incidence and prognosis, but its role in the coronavirus disease 2019 (COVID-19) pandemic remains unclear, although limited evidence has suggested a possible association [14-17]. Thus, further studies are needed to investigate associations between socioeconomic status and novel infectious diseases, as inequalities in health outcomes could be particularly evident during the COVID-19 pandemic. Therefore, this study aimed to investigate the associations between socioeconomic status, using health insurance type as a proxy, and infection with the severe acute respiratory syndrome coronavirus 2 (SARS-CoV-2) and COVID-19-related clinical outcomes.

## MATERIALS AND METHODS

### Data source

We used Korea’s Health Insurance Review and Assessment Service (HIRA) database, which was released by the Korean government as the world’s first de-identified COVID-19 nationwide data set, on March 27, 2020 (Supplementary Material 1). Korea has a universal single-payer healthcare system, provided through the NHI, that covers the entire population of 50 million. Moreover, Korea uses a fee-for-service reimbursement system that allows for healthcare utilization by people from all settings, being available for inpatients, outpatients, and nursing homes.

The HIRA database contains healthcare utilization data for all individuals who received a test for COVID-19 (as of May 15, 2020), which are also linked to their 3-year medical history (January 1, 2017 to May 15, 2020). The HIRA database anonymized all patient identifiers such that they were de-identifiable, and linked them to their socio-demographic characteristics, healthcare utilization history, diagnosis (International Classification of Diseases 10th revision codes), and prescription information. The overall positive predictive value between diagnoses recorded in claims and hospitals’ electronic medical records was found to be 82% [18]. Information on whether a patient tested positive for COVID-19 or died was linked from Korea’s Centers for Disease Control and Prevention (KCDC) database.

### Study population

**Figure 1.**
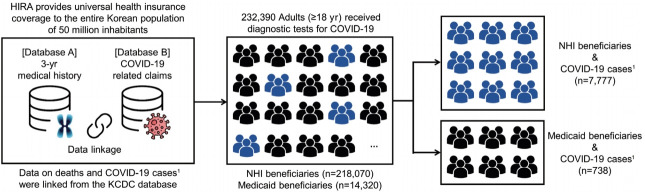
Overall diagram of our nationwide study. COVID-19, coronavirus disease 2019; HIRA, Health Insurance Review and Assessment Service; NHI, National Health Insurance; KCDC, Korea Centers for Disease Control and Prevention. ^1^Confirmed COVID-19 cases were patients with positive test results obtained from the reverse-transcription polymerase chain reaction method targeting the RNA-dependent RNA polymerase, N, and E genes.

To examine the association between health insurance type and infection with SARS-CoV-2, we identified all adults (aged ≥ 18 years) who received a diagnostic test for COVID-19 between January 1, 2020 and May 15, 2020 (n= 232,390). Among those who obtained positive results from the diagnostic test, we assessed the association between their health insurance type and the risk of COVID-19-related clinical outcomes (Figure 1). Patients were defined as confirmed cases of COVID-19 when results from reverse-transcription polymerase chain reaction tests were positive for SARS-CoV-2 RNA [19]. We classified the subjects of our study as NHI or Medicaid beneficiaries, with cohort entry defined as the earliest date of COVID-19-related claims recorded in the HIRA database (Supplementary Material 1).

### Outcome definition

We first estimated the incidence of positive COVID-19 tests among those who received a diagnostic test for COVID-19. We then estimated the incidence of COVID-19-related clinical outcomes, which were defined as a composite endpoint of all-cause death, intensive care unit (ICU) admission, and use of mechanical ventilation (primary composite outcome). We defined secondary outcomes as the individual events of the primary composite outcome (Supplementary Material 2).

### Potential confounders

We identified potential confounders by investigating their associations with the exposure (health insurance type) and outcome (COVID-19 incidence or its subsequent clinical outcomes). Age and sex were assessed on cohort entry. Comorbidities (hypertension, hyperlipidemia, diabetes mellitus, asthma, chronic obstructive pulmonary disease, atherosclerosis, heart failure, myocardial infarction, stroke, renal failure, chronic liver disease, fractures, osteoarthritis, rheumatoid arthritis, psychiatric disorders, thyroid disorders, osteoporosis, dementia, malignancy), severe incurable diseases (defined based on Medicaid eligibility criteria, including rare or incurable diseases), and use of co-medications (angiotensin converting enzyme inhibitors, angiotensin-receptor II blockers, β-blockers, calcium channel blockers, diuretics, nitrates, antidiabetic medications including insulin, anxiolytics, antipsychotics, antidepressants, non-steroidal anti-inflammatory drugs [NSAIDs], anticoagulants) were assessed in the year prior to cohort entry (Supplementary Material 2). We also calculated the Charlson comorbidity index (CCI) score using previously validated algorithms [20,21].

### Statistical analysis

We summarized patients’ baseline characteristics using counts with proportions for categorical variables or mean values with standard deviation (SD) for continuous variables. We used the chi-square test for categorical variables and the t-test for continuous variables to determine whether any statistically significant differences were present between health insurance types.

We estimated the cumulative incidence with 95% confidence intervals (CIs) for infection with SARS-CoV-2 and COVID19-related clinical outcomes for each health insurance type. We then constructed 3 logistic regression models to estimate the odds ratio (OR) with 95% CIs for the risk of SARS-CoV-2 infection or the risk of adverse clinical outcomes associated with health insurance type. The first model was unadjusted for potential confounders. The second model was adjusted for age and sex. The third model, which we considered our main analysis, was adjusted for age, sex, and the CCI score.

### Subgroup and sensitivity analyses

We conducted subgroup analyses for both study outcomes (SARS-CoV-2 infection and COVID-19-related clinical outcomes) by stratifying according to Medicaid type (type 1 or type 2), sex, and age group (< 60, 60-69, 70-79, or ≥ 80 years). In Korea, Medicaid beneficiaries, or those who earn ≤ 40% of the national median household income, are classified into either type 1 (individuals who are incapable of working) or type 2 (those who are capable of working) [9].

For the sensitivity analysis, we estimated propensity scores (PS) using multivariable logistic regression analysis to obtain comparability between beneficiaries of NHI or Medicaid programs. The health insurance type was set as the dependent variable and all confounders that had a possible association (p< 0.2 in the univariate analysis) with the outcome were included as independent variables [22]; age, sex, and CCI score were always included in the model, regardless of their p-values. Using the estimated PS, we applied inverse probability of treatment (IPT) weights; in this instance, the treatment was the type of health insurance [23,24]. We then conducted univariable logistic regression analyses to estimate IPT-weighted ORs with 95% CIs for COVID-19 incidence or COVID-19-related clinical outcomes associated with the health insurance type.

All statistical analyses were performed using the SAS version 9.4 (SAS Institute Inc., Cary, NC, USA), in which a 2-tailed alpha of 0.05 was considered to indicate statistical significance.

### Ethics statement

Our study complies with the Declaration of Helsinki and the study protocol was approved by the Institutional Review Board of Sungkyunkwan University (SKKU 2020-03-012); the requirement to obtain informed consent was waived by the board.

## RESULTS

Of the 232,390 individuals who received a diagnostic test for COVID-19, 218,070 (93.8%) and 14,320 (6.2%) were beneficiaries of NHI and Medicaid, respectively (Figure 1). Compared to NHI, Medicaid beneficiaries were older (mean age, 63.8± 17.7 years vs. 50.2± 20.0; p< 0.001), had a higher male percentage (51.5 vs. 47.2%; p< 0.001), and had a higher comorbidity burden (mean CCI score, 2.4± 1.6 vs. 2.2± 1.7; p<0.001). Medicaid beneficiaries had a higher prevalence of comorbidity history and use of co-medications than NHI beneficiaries (Table 1).

Of the individuals who received COVID-19 tests, 8,515 were confirmed cases of COVID-19, of whom 7,777 (91.3%) were NHI beneficiaries and 738 (8.7%) were Medicaid beneficiaries (Figure 1). Among the COVID-19 cases, Medicaid beneficiaries, as compared with NHI beneficiaries, were also older (mean age, 57.5± 16.8 vs. 47.8± 19.1; p< 0.001), had a higher proportion of males (47.2 vs. 40.2%; p< 0.001), and had a higher comorbidity burden (mean CCI score, 2.0± 1.1 vs. 1.7± 1.0; p< 0.001). Except for the use of NSAIDs (71.4 vs. 78.4%; p< 0.001), Medicaid beneficiaries had a comparable or higher prevalence of comorbidity history and use of co-medications than NHI beneficiaries (Table 1).

**Figure 2.**
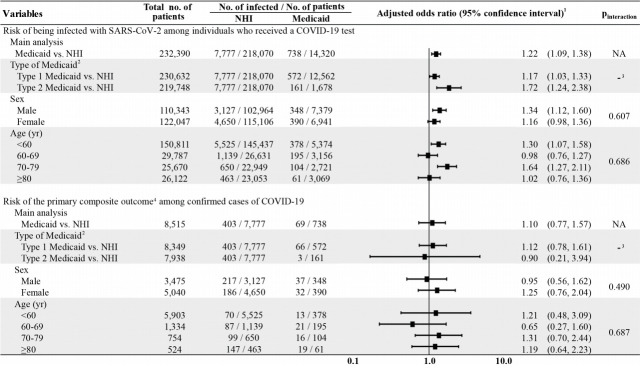
Forest plot summarizing the risk of being infected with SARS-CoV-2 and the risk of the primary composite outcome associated with health insurance type when stratified for Medicaid type, sex, and age group. COVID-19, coronavirus disease 2019; NA, not applicable; NHI, National Health Insurance, SARS-CoV-2, severe acute respiratory syndrome coronavirus 2. ^1^Adjusted for age, sex, Charlson comorbidity index score in the multivariable logistic regression model. ^2^Medicaid beneficiaries, or those who earn <40% of the national median household income, are classified into either type 1 (individuals who are incapable of working) or type 2 (those who are capable of working) in Korea. ^3^P-for-interaction was not calculated, as the subtype of Medicaid was an exposure variable. ^4^The primary composite outcome included all-cause death, intensive care unit admission, and mechanical ventilation use.

The cumulative incidence of SARS-CoV-2 infection was 5.15% (738 out of 14,320) and 3.57% (7,777 out of 218,070) for Medicaid and NHI beneficiaries, respectively. Compared to NHI beneficiaries, Medicaid beneficiaries were associated with a 22% higher risk of SARS-CoV-2 infection (age-, sex-, CCI-adjusted OR, 1.22; 95% CI, 1.09 to 1.38); the sensitivity analysis results remained consistent (IPT-weighted OR, 1.17; 95% CI, 1.05 to 1.30) (Table 2). The findings from our subgroup analyses revealed no statistically significant differences in the associations of SARS-CoV-2 infection by Medicaid type, sex, and age group (Figure 2).

For the primary composite outcome (all-cause death, ICU admission, mechanical ventilation use), the cumulative incidence was 9.35% (69 out of 738) and 5.18% (403 out of 7,777) for Medicaid and NHI beneficiaries, respectively. Compared to NHI beneficiaries, Medicaid beneficiaries had no significantly distinct association with the primary composite outcome (age-, sex-, CCI-adjusted OR, 1.10; 95% CI, 0.77 to 1.57). In assessing the individual events of the primary composite outcome, Medicaid beneficiaries—as compared with NHI beneficiaries—did not show an increased risk of all-cause death (OR, 1.35; 95% CI, 0.90 to 2.02), ICU admission (OR, 0.98; 95% CI, 0.53 to 1.79), or mechanical ventilation use (OR, 0.77; 95% CI, 0.41 to 1.42), and the sensitivity analysis yielded analogous findings (Table 2). Likewise, there was no association between Medicaid beneficiaries and the risk of our primary composite outcome by Medicaid type, sex, and age group (Figure 2).

## DISCUSSION

In this nationwide retrospective cohort study of 14,320 patients who received diagnostic tests for COVID-19 in Korea, Medicaid beneficiaries, as compared with NHI beneficiaries, had a 22% higher risk of infection with SARS-CoV-2. However, Medicaid beneficiaries had non-statistically significant associations with the risk of our primary composite outcome of all-cause death, ICU admission, and mechanical ventilation use, when compared to NHI beneficiaries. Thus, our findings suggest that although Medicaid beneficiaries had higher risks of being infected with SARS-CoV-2, the risk of COVID-19-related clinical outcomes was not significantly different. The findings of this study provide important and novel evidence that, during times of infectious disease pandemics, disparities in socioeconomic status could further heighten this gap as Medicaid beneficiaries were more susceptible to COVID-19 than NHI beneficiaries.

This study reconfirms the vital role that socioeconomic status may have regarding the incidence of novel communicable diseases and their consequences, in contrast to previous studies that mainly focused on non-communicable chronic diseases or communicable non-respiratory diseases [10,25-27]. Although those studies proposed traditional socioeconomic-related mechanisms such as unsanitary problems or deprived environments regarding the risk of infectious diseases [7,8], our study raises an alternative possibility in that socioeconomic factors may also serve as determinants or as transmission mechanisms in infectious diseases. COVID-19 appears to function as a new path to further widen the health disparities stemming from socioeconomic inequalities present in our society. However, regarding the subsequent clinical outcomes, socioeconomic inequalities, using health insurance type as a proxy, were unlikely to affect the gap in healthcare accessibility in medical institutions, which is an inconsistent result when compared to prior findings [3,10-13]. Moreover, such estimates were determined by comparing those with the highest income among NHI beneficiaries, a group that is considered to be the most different from those benefitting from Medicaid. Furthermore, the work capability of individuals, as a key component of socioeconomic status, is less likely to impact the clinical outcomes of COVID-19 among Medicaid beneficiaries with low socioeconomic status. Nevertheless, in an epidemic crisis with severe propagation power, such as COVID-19, our findings could serve as evidence to support the well-operating public health system and medical institutions in Korea, regardless of patients’ socioeconomic background.

Our findings underscore the need to clarify priorities in healthcare policies, although it still remains unclear as to which specific socioeconomic factor mainly impacts the risk of being infected with SARS-CoV-2 among environmental vulnerability to exposure, health behavior patterns, and communication patterns of population groups with low socioeconomic status. Previous studies have reported that Medicaid beneficiaries had higher risks for lower levels of medication adherence and treatment persistence, lower effectiveness of behavioral intervention programs, and greater vulnerability to environmental conditions [28]. In support of these findings, the Medicaid beneficiaries in our study were older, had a higher prevalence of comorbidity history and use of co-medications, and had a higher increased risk of SARS-CoV-2 infection than NHI beneficiaries. Thus, pre-emptive support should be provided first and foremost to high-risk groups as they are most likely to be impacted during times of economic or epidemic crises as a result of their vulnerable socioeconomic status, and further, to slow or eliminate the spread of COVID-19.

The major strength of this study is that we used a nationwide healthcare database of Korea, with representativeness of the entire Korean population, that includes information on healthcare utilization of all COVID-19-related claims as of May 15, 2020. Thus, our findings provide real-world evidence that could prove useful in shaping future healthcare policies to narrow the currently prevalent socioeconomic disparities in times of epidemics or pandemics of infectious diseases. In addition, the HIRA database used in this study is the world’s first open and de-identified database containing nationwide information of patients with COVID-19. In being open to both domestic and international researchers, our findings are believed to have high reproducibility. With its source population, our data set was sufficiently large to assess this socially important issue.

Our study has some limitations. First, outcome misclassification is possible in defining ICU admission or mechanical ventilation use. However, the validity of the national procedure codes used to define these outcomes is believed to be high as these codes are used for reimbursement processes by the Korean health insurance authority. Meanwhile, misclassification of a positive test result for COVID-19 or all-cause death is likely not to have occurred in our study as these records were linked to those of the KCDC, which have been thoroughly reviewed by numerous clinicians and government officials. Second, we did not have access to data on details of socioeconomic status, such as income; occupation; lifestyle factors, such as obesity, alcohol consumption, or smoking status; or a comprehensive range of factors that could be associated with worsened clinical outcomes. Last, residual confounding from unmeasured confounders may be present due to the inherent limitations of the health insurance claims-related data used in this study. Nevertheless, by using health insurance type, which takes into account an individual’s income level, occupation, and various other factors, as a surrogate measure for socioeconomic status, we were able to demonstrate well our study objectives of assessing the association between socioeconomic status and infection with SARS-CoV-2, as well as COVID-19-related clinical outcomes.

In conclusion, the findings of this nationwide retrospective cohort study indicate that socioeconomic status, using health insurance type as a proxy, was associated with a higher risk of infection with SARS-CoV-2. However, we found no association between socioeconomic status and the risk of a composite endpoint of allcause death, ICU admission, and mechanical ventilation use. In the meantime, although further investigations are warranted, preemptive support should be provided to high-risk groups, such as Medicaid beneficiaries, to slow or possibly eliminate the spread of COVID-19 during the ongoing pandemic.

## Figures and Tables

**Table 1.** Characteristics of individuals who received COVID-19 diagnostic tests and those who tested positive, by health insurance type, in Korea

Characteristics	Recipients of COVID-19 diagnostic tests (n=232,390)			Confirmed cases of COVID-19 (n=8,515)		
	NHI (n=218,070)	Medicaid (n=14,320)		NHI (n=7,777)	Medicaid (n=738)	p-value^1^
Age (yr)^2^						
Mean±SD	50.2±20.0	63.8±17.7	<0.001	47.8±19.1	57.5±16.8	<0.001
19-29	41,802 (19.2)	840 (5.9)	<0.001	2,039 (26.2)	79 (10.7)	<0.001
30-39	39,995 (18.3)	541 (3.8)		903 (11.6)	17 (2.3)	
40-49	33,040 (15.2)	1,355 (9.5)		1,047 (13.5)	87 (11.8)	
50-59	30,600 (14.0)	2,638 (18.4)		1,536 (19.8)	195 (26.4)	
60-69	26,631 (12.2)	3,156 (22.0)		1,139 (14.6)	195 (26.4)	
70-79	22,949 (10.5)	2,721 (19.0)		650 (8.4)	104 (14.1)	
80-89	19,257 (8.8)	2,389 (16.7)		391 (5.0)	48 (6.5)	
>89	3,796 (1.7)	680 (4.7)		72 (0.9)	13 (1.8)	
Sex^2^			<0.001			<0.001
Male	102,964 (47.2)	7,379 (51.5)		3,127 (40.2)	348 (47.2)	
Female	115,106 (52.8)	6,941 (48.5)		4,650 (59.8)	390 (52.8)	
Charlson comorbidity index score^3^						
Mean±SD	2.2±1.7	2.4±1.6	<0.001	1.7±1.0	2.0±1.1	<0.001
1	37,850 (17.4)	3,227 (22.5)	<0.001	1,229 (15.8)	91 (12.3)	<0.001
2	29,667 (13.6)	3,305 (23.1)		862 (11.1)	165 (22.4)	
≥3	24,905 (11.4)	3,802 (26.6)		387 (5.0)	65 (8.8)	
Comorbidities^3^						
Hypertension	48,930 (22.4)	5,999 (41.9)	<0.001	1,372 (17.6)	208 (28.2)	<0.001
Hyperlipidemia	38,384 (17.6)	3,852 (26.9)	<0.001	1,224 (15.7)	183 (24.8)	<0.001
Diabetes mellitus	29,495 (13.5)	4,470 (31.2)	<0.001	777 (10.0)	145 (19.6)	<0.001
Asthma	15,375 (7.1)	1,761 (12.3)	<0.001	383 (4.9)	33 (4.5)	0.585
COPD	37,010 (17.0)	3,694 (25.8)	<0.001	1,021 (13.1)	84 (11.4)	0.177
Atherosclerosis	2,161 (1.0)	331 (2.3)	<0.001	31 (0.4)	11 (1.5)	<0.001
Heart failure	5,282 (2.4)	942 (6.6)	<0.001	84 (1.1)	17 (2.3)	0.003
Stroke	9,384 (4.3)	1,741 (12.2)	<0.001	198 (2.5)	61 (8.3)	<0.001
Myocardial infarction	1,565 (0.7)	262 (1.8)	<0.001	32 (0.4)	6 (0.8)	0.118
Renal failure	8,091 (3.7)	1,792 (12.5)	<0.001	56 (0.7)	17 (2.3)	<0.001
Chronic liver disease	16,894 (7.7)	2,175 (15.2)	<0.001	457 (5.9)	83 (11.2)	<0.001
Fracture	14,394 (6.6)	2,080 (14.5)	<0.001	344 (4.4)	62 (8.4)	<0.001
Osteoarthritis	33,865 (15.5)	4,131 (28.8)	<0.001	1,063 (13.7)	159 (21.5)	<0.001
Rheumatoid arthritis	3,152 (1.4)	315 (2.2)	<0.001	105 (1.4)	9 (1.2)	0.768
Psychiatric disorders	33,143 (15.2)	6,133 (42.8)	<0.001	857 (11.0)	386 (52.3)	<0.001
Thyroid disorders	11,993 (5.5)	1,067 (7.5)	<0.001	389 (5.0)	52 (7.0)	0.017
Osteoporosis	11,052 (5.1)	1,533 (10.7)	<0.001	413 (5.3)	54 (7.3)	0.022
Dementia	11,885 (5.5)	2,253 (15.7)	<0.001	315 (4.1)	77 (10.4)	<0.001
Cancer	21,039 (9.6)	1,834 (12.8)	<0.001	253 (3.3)	31 (4.2)	0.171
Severe incurable disease^4^	37,628 (17.3)	4,429 (30.9)	<0.001	495 (6.4)	60 (8.1)	0.063
Concomitant medications^3^						
ACE inhibitors	2,427 (1.1)	396 (2.8)	<0.001	68 (0.9)	12 (1.6)	0.043
ARBs	40,853 (18.7)	5,341 (37.3)	<0.001	1,096 (14.1)	172 (23.3)	<0.001
β-blockers	28,367 (13.0)	4,272 (29.8)	<0.001	667 (8.6)	171 (23.2)	<0.001
Calcium channel blockers	40,060 (18.4)	5,652 (39.5)	<0.001	943 (12.1)	173 (23.4)	<0.001
Diuretics	14,107 (6.5)	1,734 (12.1)	<0.001	429 (5.5)	49 (6.6)	0.205
Nitrates	8,932 (4.1)	1,469 (10.3)	<0.001	103 (1.3)	20 (2.7)	0.003
Antidiabetic drugs	28,926 (13.3)	4,401 (30.7)	<0.001	729 (9.4)	149 (20.2)	<0.001
Anxiolytics	127,676 (58.5)	12,052 (84.2)	<0.001	3,186 (41.0)	544 (73.7)	<0.001
Antipsychotics	16,336 (7.5)	4,494 (31.4)	<0.001	316 (4.1)	292 (39.6)	<0.001
Antidepressants	30,304 (13.9)	5,289 (36.9)	<0.001	721 (9.3)	231 (31.3)	<0.001
NSAIDs	177,366 (81.3)	11,814 (82.5)	<0.001	6,101 (78.4)	527 (71.4)	<0.001
Anticoagulants	53,794 (24.7)	7,739 (54.0)	<0.001	1,149 (14.8)	231 (31.3)	<0.001

Values are presented as number (%). COVID-19, coronavirus disease 2019; NHI, National Health Insurance; SD, standard deviation; COPD, chronic obstructive pulmonary disease; ACE, angiotensin converting enzyme; ARB, angiotensin-receptor II blocker; NSAID, non-steroidal anti-inflammatory drug.

1 The chi-square test for categorical variables and the t-test for continuous variables were used to determine statistically significant differences between health insurance types.

2 Assessed on cohort entry (the date when subjects received the test for COVID-19 or the date when subjects tested positive for COVID-19).

3 Assessed in the year prior to cohort entry.

4 Severe incurable diseases are Medicaid eligibility criteria, which include rare or incurable diseases.

**Table 2.** Risk of SARS-CoV-2 infection among individuals who received a COVID-19 diagnostic test or poor clinical outcomes among positive cases of COVID-19, by health insurance type

Characteristics	No. of subjects	No. of events	No. of events per 100 patients, % (95% CI)	Unadjusted model	Age- and sex-adjusted model	IPT weighted model^1,2^	Age-, sex-, CCI- adjusted model
Risk of SARS-CoV-2 infection							
NHI	218,070	7,777	3.57 (3.49, 3.64)	1.00 (reference)	1.00 (reference)	1.00 (reference)	1.00 (reference)
Medicaid	14,320	738	5.15 (4.79, 5.52)	1.47 (1.36, 1.59)	1.54 (1.42, 1.67)	1.17 (1.05, 1.30)	1.22 (1.09 1.38)
Primary composite outcome							
NHI	7,777	403	5.18 (4.69, 5.67)	1.00 (reference)	1.00 (reference)	1.00 (reference)	1.00 (reference)
Medicaid	738	69	9.35 (7.25, 11.45)	1.89 (1.45, 2.47)	1.26 (0.95, 1.67)	1.20 (0.90, 1.60)	1.10 (0.77, 1.57)
All-cause death							
NHI	7,777	238	3.06 (2.68, 3.44)	1.00 (reference)	1.00 (reference)	1.00 (reference)	1.00 (reference)
Medicaid	738	51	6.91 (5.08, 8.74)	2.35 (1.72, 3.21)	1.68 (1.19, 2.36)	1.31 (0.95, 1.80)	1.35 (0.90, 2.02)
Intensive care unit admission							
NHI	7,777	171	2.20 (1.87, 2.52)	1.00 (reference)	1.00 (reference)	1.00 (reference)	1.00 (reference)
Medicaid	738	18	2.44 (1.33, 3.55)	1.11 (0.68, 1.82)	0.74 (0.45, 1.21)	1.35 (0.84, 2.16)	0.98 (0.53, 1.79)
Mechanical ventilation use							
NHI	7,777	166	2.13 (1.81, 2.46)	1.00 (reference)	1.00 (reference)	1.00 (reference)	1.00 (reference)
Medicaid	738	22	2.98 (1.75, 4.21)	1.41 (0.90, 2.21)	0.86 (0.55, 1.37)	0.96 (0.58, 1.57)	0.77 (0.41, 1.42)

Values are presented as odds ratio (95% confidence interval). SARS-CoV-2, severe acute respiratory syndrome coronavirus 2; COVID-19, coronavirus disease 2019; IPT, inverse probability of treatment; CCI, Charlson comorbidity index score; NHI, National Health Insurance.

1 IPT-weighted multivariable logistic regression model (considered our sensitivity analysis) for the risk of SARS-CoV-2 infection, in which the propensity score was estimated by including age, sex, CCI, hypertension, hyperlipidemia, diabetes mellitus, asthma, chronic obstructive pulmonary disease, atherosclerosis, heart failure, myocardial infarction, stroke, renal failure, chronic liver disease, fractures, osteoarthritis, psychiatric disorders, thyroid disorders, dementia, malignancy, severe incurable diseases, angiotensin converting enzyme inhibitors, angiotensin-receptor II blockers, β-blockers, calcium channel blockers, diuretics, nitrates, antidiabetic medications including insulin, anxiolytics, antipsychotics, antidepressants, non-steroidal anti-inflammatory drugs, and anticoagulants in the multivariable logistic regression model (c-statistic, 0.719).

2 IPT-weighted multivariable logistic regression model (considered our sensitivity analysis) for the risk of worsened clinical outcomes, in which the propensity score was estimated by including age, CCI, hypertension, hyperlipidemia, diabetes mellitus, asthma, chronic obstructive pulmonary disease, atherosclerosis, heart failure, myocardial infarction, renal failure, chronic liver disease, fractures, osteoarthritis, psychiatric disorders, osteoporosis, dementia, malignancy, severe incurable diseases, and use of angiotensin converting enzyme inhibitors, angiotensin-receptor II blockers, β-blockers, calcium channel blockers, diuretics, nitrates, antidiabetic medications including insulin, anxiolytics, antipsychotics, antidepressants, and anticoagulants in the multivariable logistic regression model (c-statistic, 0.779).

## Supplementary Materials

Supplementary Material 1. Schema of the Health Insurance Review and Assessment Service data

Supplementary Material 2. Diagnosis and drug codes used in the study
