# Supplementary material for: Socioeconomic disparities in Korea by health insurance type during the COVID-19 pandemic: a nationwide study
Source: Epidemiol Health. 2021 Jan 13;43:e2021007. doi: 10.4178/epih.e2021007 (PMC8060526; doi:10.4178/epih.e2021007)
Supplement: Supplementary Material 1. — Schema of the Health Insurance Review and Assessment Service data [file epih-43-e2021007-suppl1.pdf]

Volume: 43, Article ID: e2021007

https://doi.org/10.4178/epih.e2021007

Supplementary Material 1. Schema of the Health Insurance Review and Assessment Service data[†]

Categorization	Table Name	Variables Included and their Description
Subjects who received tests for COVID-19[‡]	Sociodemographic table	Sex, age, region of residence, medical institution type, others
	Healthcare utilization table	Date of visit, duration of hospital stay, expenditure, others
	Diagnosis table	ICD-10 diagnosis codes, specialty, others
	Prescription table	National drug chemical code, day's supply, dosage, others
3-year medical history of subjects who received tests for COVID-19[¶]	Sociodemographic table	Sex, age, region of residence, medical institution type, others
	Healthcare utilization table	Date of visit, duration of hospital stay, expenditure, others
	Diagnosis table	ICD-10 diagnosis codes, specialty, others
	Prescription table	National drug chemical code, day's supply, dosage, others

Note: ICD-10, International Classification of Disease 10th Revision

[†]In the nationwide patient Big Data of Health Insurance Review and Assessment Service of Korea, provided as part of the COVID-19 International Research of South Korea, there are eight data tables. For more information, please refer to the "Data Schema" file at https://hira-covid19.net/

[‡]Data extraction criteria: COVID-19 related claims with 1) "3/02" in classification code of MT043 (target of medical cost support due to national disaster) based on the serial number of claim statement, 2) "D6584" COVID-19 (real-time polymerase chain reaction), 3) COVID-19 related diagnosis codes (KCD-7: B342, B972, Z208, Z290, U18, U181, Z038, Z115, U071, U072), or 4) other COVID-19 related reimbursement codes (COVID-19 related admission fee, management fee, IUD, isolation, public relief hospital, residential treatment center, screening center, negative pressure room, others)

[¶]Data extraction criteria: healthcare service use claims from January 1, 2017 to May 15, 2020 of patients whose had a claim for the above-mentioned COVID-19 related criteria
