# Supplementary material for: Socioeconomic disparities in Korea by health insurance type during the COVID-19 pandemic: a nationwide study
Source: Epidemiol Health. 2021 Jan 13;43:e2021007. doi: 10.4178/epih.e2021007 (PMC8060526; doi:10.4178/epih.e2021007)
Supplement: Supplementary Material 2. — Diagnosis and drug codes used in the study [file epih-43-e2021007-suppl2.pdf]

Volume: 43, Article ID: e2021007

https://doi.org/10.4178/epih.e2021007

Supplementary Material 2. Diagnosis and drug codes used in the study

Type	Name	Codes
Diagnoses		
COVID-19 (KCD-7)		B34.2, B92. 7, Z20.8, Z29.0, U18, U18.1, Z03.8, Z11.5, U07.1, U07.2
Study Outcomes	Death (ICD-10)	Y/N, I46.1, I46.9, R96, R98, R99
	Intensive care unit (NPC)	AH190, AH290, AH390, AH110, AH210, AJ001, AJ003, AJ004, AJ005, AJ006, AJ007, AJ008, AJ009, AJ100, AJ200, AJ101, AJ102, AJ201, AJ202
	Mechanical ventilation (NPC)	M0850, M0857, M0858, M0860, M5830, M5850, M5857, M5858, M5860, MM360, MM400
Comorbidities (ICD-10)	Hypertension	I10-I15
	Hyperlipidemia	E78
	Diabetes mellitus	E10-E14
	Asthma	J45, J46
	COPD	J40-J44
	Atherosclerosis	I25
	Heart failure	I50
	Myocardial infarction	I21
	Stroke	I60-I64
	Renal failure	N17-N19
	Chronic liver disease	K70, K71.3-K71.5, K73, K74, K76
	Fracture	S02 (excl. S02.5), S12, S22, S32, S42, S52, S62, S72, S82, S92, T02, T08, T10, T12, M48.4, M48.5, M84.3
	Osteoarthritis	M15-M19
	Rheumatoid arthritis	M05-M06
	Psychiatric disorders	F04-F10, F20-F99
	Thyroid disorders	E01-E03, E05-E07
	Osteoporosis	M80-M82
	Dementia	F00-F03, G30, G31.00, G31.82
Serious incurable diseases (expanded benefit coverage codes)		V000-V206
Drugs (ATC)		
Co-medications	ACE inhibitors	C09A
	Angiotensin receptor II blockers	C09C
	β-blockers	C07
	Calcium channel blockers	C08
	Diuretics	C03
	Nitrates	C01DA
	Antidiabetic medications (incl. insulin)	A10
	Anxiolytics	N05B
	Antipsychotics	N05A
	Antidepressants	N06A
	NSAIDs	M01
	Anticoagulants	B01A

Note: ACE, angiotensin converting enzyme; ATC, Anatomical Therapeutic Chemical; COPD, chronic obstructive pulmonary disease; KCD-7, Korean Standard Classification of Diseases, 7th Revision; ICD-10, International Classification of Diseases, 10th Revision, NPC, national procedure codes; NSAIDs, nonsteroidal anti-inflammatory drugs
